# ENCODE data at the ENCODE portal

**DOI:** 10.1093/nar/gkv1160

**Published:** 2015-11-02

**Authors:** Cricket A. Sloan, Esther T. Chan, Jean M. Davidson, Venkat S. Malladi, J. Seth Strattan, Benjamin C. Hitz, Idan Gabdank, Aditi K. Narayanan, Marcus Ho, Brian T. Lee, Laurence D. Rowe, Timothy R. Dreszer, Greg Roe, Nikhil R. Podduturi, Forrest Tanaka, Eurie L. Hong, J. Michael Cherry

**Affiliations:** 1 Stanford University School of Medicine, Department of Genetics, Stanford, CA, 94305, USA; 2 University of California at Santa Cruz, Center for Biomolecular Science and Engineering, Santa Cruz, CA, 95064, USA

## Abstract

The Encyclopedia of DNA Elements (ENCODE) Project is in its third phase of creating a comprehensive catalog of functional elements in the human genome. This phase of the project includes an expansion of assays that measure diverse RNA populations, identify proteins that interact with RNA and DNA, probe regions of DNA hypersensitivity, and measure levels of DNA methylation in a wide range of cell and tissue types to identify putative regulatory elements. To date, results for almost 5000 experiments have been released for use by the scientific community. These data are available for searching, visualization and download at the new ENCODE Portal (www.encodeproject.org). The revamped ENCODE Portal provides new ways to browse and search the ENCODE data based on the metadata that describe the assays as well as summaries of the assays that focus on data provenance. In addition, it is a flexible platform that allows integration of genomic data from multiple projects. The portal experience was designed to improve access to ENCODE data by relying on metadata that allow reusability and reproducibility of the experiments.

## INTRODUCTION

The Encyclopedia of DNA Elements (ENCODE) Project began as a Pilot Project on 1% of the human genome (1,2). In 2007, the effort was scaled to whole-genome assays followed by expansion to performing similar assays in mouse (3–5). The ENCODE Project continues to create a comprehensive catalog of gene elements and functional elements in the human and mouse genomes by measuring RNA expression levels, identifying proteins that interact with RNA and DNA (such as modified histones, transcription factors and RNA-binding proteins), measuring the levels of DNA methylation and identifying regions of DNA hypersensitivity. These data generated by ENCODE Consortium members are submitted to the ENCODE Data Coordination Center (DCC) whose primary task is to curate, process and validate the data in preparation for release to the scientific community. To promote the sharing of data, the ENCODE consortium has updated its release policy (https://www.encodeproject.org/about/data-use-policy/). These data are made available at the ENCODE Portal (www.encodeproject.org), created by the DCC, and are also distributed through the UCSC genome browser, GEO and Ensembl and used extensively as a community resource (6–11).

To increase the reproducibility, reusability and interoperability of the ENCODE data, the ENCODE DCC, in collaboration with the ENCODE consortium, has defined a new metadata standard. The experimental assays and computational methods used to generate the ENCODE data are represented by a structured data model to capture metadata that allows for maximal understanding and interpretation of these results (Hong *et al*., submitted). Integral to the metadata, persistent identifiers are assigned for experiments, files, antibody lots and biosamples to allow specific identification of the data. In addition, controlled vocabularies and ontologies are used to increase the interoperability of the ENCODE data with other projects (12).

Specifically, the experiment metadata displayed on the portal utilize the Uber Anatomy Ontology (UBERON, uberon.org) for tissue biosamples; Cell Ontology (CL, cellontology.org) for primary cells; Experimental Factor Ontology (EFO, www.ebi.ac.uk/efo/) for immortalized cell lines; Ontology for Biomedical Investigations (OBI, obi-ontology.org) for experimental assays; Chemical Entities of Biological Interest (ChEBI, www.ebi.ac.uk/chebi/) for chemical treatments; and Sequence Ontology (SO, sequenceontology.org) for the nucleic acids of interest.

As discussions about the issues of reproducibility and reusability of genomic data are becoming more significant (13,14), the new ENCODE Portal serves to communicate to the scientific community the experimental standards and guidelines defined by the entire Consortium, as well as the details of how the assays were performed and the computational methods used to analyze the data.

## NEW ENCODE DATA PORTAL

**Figure 1.**
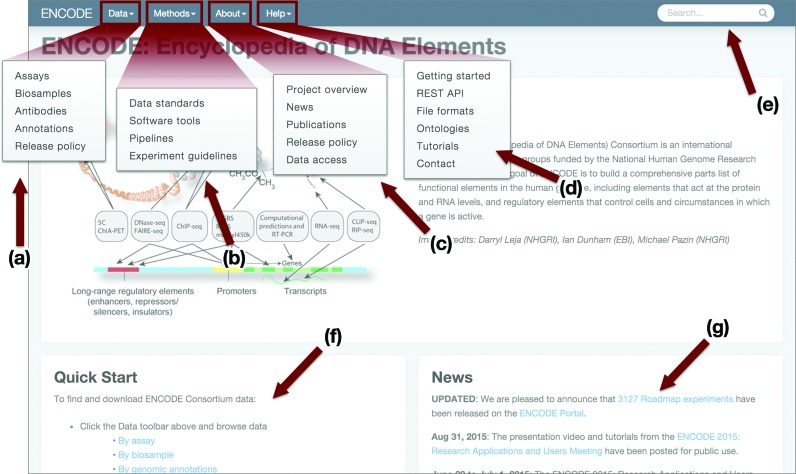
Overview of the ENCODE Portal ‘dashboard-style’ home page. (**a**) The Data section provides links into search pages that allow filtering of the ENCODE data. (**b**) The Methods section contains standard documents, software and analysis pipeline information. (**c**) The About ENCODE section provides context to the ENCODE project, the site and access to relevant publications. (**d**) The Help section lists tutorials and guide pages. (**e**) The free-text search box for querying any metadata or text item. (**f**) The Quick Start guide has popular links to browsing and download information. (**g**) The News feed tracks updates and data released. Items (a) to (e) are part of a persistent menu bar that appears on every page to provide stable navigation throughout the site.

The new ENCODE data portal, released in August 2014, is the canonical, central source for ENCODE raw data, analysis data, methods, standards and experimental metadata. The user experience begins with a dashboard-style homepage (Figure 1), that includes a news feed, menus and a quick start guide providing instruction on the various ways to access the data via browsing, searching, visualizing and downloading. A persistent standard menu at the top of every page provides stable navigation throughout the site. Within the menu are the categories of ‘Data’, ‘Methods’, ‘About ENCODE’ and ‘Help’. The ‘Data’ section provides initial search access into the data as well as a link to the new Release Policy that describes the terms of data use. The primary access to the ENCODE data is through the ‘Assays’ search page. This page allows one to browse replicated experimental sets, by faceted search selection on the left to filter experiments by features such as assay type, biosample type, organism or treatment. The biosample page provides similar browsing access to a listing of all individual biosamples used in the experiments. The antibody page provides access to the antibodies screened or used in the ENCODE immunoprecipitation experiments along with the required antibody characterizations generated by the project (15). The annotation section links publications and analysis files generated by the consortium towards the goal of creating an encyclopedia of DNA elements. This section also contain links to multiple browsers to view annotations created from collations of information from the many ENCODE experiments.

The ‘Methods’ section includes information on the data standards, software, analysis pipelines and experimental guidelines defined by the consortium. The ENCODE consortium develops internal standards on the quality and procedures for both the experimental methods and the computational analysis. Current standards and guidelines are available from the portal as well as an archive containing previous versions. Additionally, a large variety of software is used in the analysis of these datasets. To improve transparency, the ENCODE Portal is hosting version information and links to source code for the software used in ENCODE project analysis. The goal is to provide sufficient information to allow anyone in the community to easily repeat the analysis if so desired. The ENCODE consortium is also developing uniform analysis pipelines that map raw sequencing data for various assays, generate visualizable signal files from those mappings and produce a quantification or annotation result. Summaries of the these pipelines, along with general specifications, are linked from the ‘Pipelines' page. The ‘About ENCODE’ section gives context to the wealth of experiments found on this site, providing a project overview, access to all news items and information about data access at other sites. This section also has the ‘Publications’ page which provides guided entry into browsing a curated collection of publications that either use ENCODE data or are references for the methods used in creating the ENCODE data. Finally, the ‘Help’ menu has a variety of help documents and tutorials to facilitate differing user experiences. Included in this section are instructions on how to browse the data in the UI, using the REST API programmatic download.

## DATA ACCESS FEATURES

Access to the ENCODE data from the new portal is driven by high-quality metadata that provide data provenance and transparency by describing how the assays were performed and how the data were analyzed. In order to import the data from previous phases of the ENCODE Project, a massive data review was undertaken to match files with controls, to include any information that was found in the documentation, to work with laboratory groups to bring their older metadata up to the newer standards (including capturing protocols, sequencing platforms used, replicate structure, and shared biosamples when it could be known), to assign specific ontology terms for the biosamples and capture library preparation details. With this depth and breadth of metadata, the new portal is able to provide a more advanced level of searching, a more comprehensive view of the assays performed and visualization of the ENCODE data using the selected subset of the data.

### Browsing and searching

**Figure 2.**
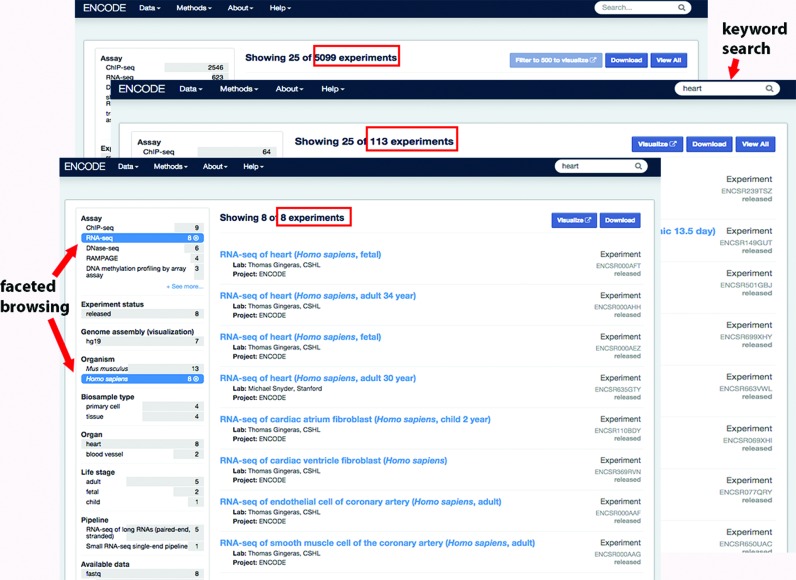
Using search and filtering to select relevant experiments. The organization of key experiment metadata into facets allows the user to quickly filter from nearly 5000 to only eight experiments with a few simple terms. Entering ‘heart’ in the search box reduced the list to 105 experimental datasets. Filtering further on the terms ‘RNA-seq’ and ‘*Homo sapiens*’ using the facets limited the list even further down to 8 experiments.

ENCODE data can be accessed both by browsing via faceted search and by searching via keywords. The faceted search page, accessible via the ‘Assay’ link underneath the ‘Data’ menu item in the toolbar, provides a simplified interface that allows the filtration of thousands of assays to the ones of interest in a few clicks. Using a few simple terms like heart, RNA-seq and *Homo sapiens* can reduce the experimental dataset list from nearly 5000 to 8 relevant experiments. The keyword search is a string search, in the upper right hand corner, for biosamples, assays, GEO identifiers, ENCODE identifiers, targets or primary investigators. Ontologies are used to further expand the search into synonyms and *derived_from* relationships. This feature allows users to enter a term like ‘heart’ or ‘K562’ into the search box and find all relevant biosamples, targets and experiments. The results of this search can be further filtered by the aforementioned faceted search (Figure 2). Both keyword search and faceted search are driven by our rich metadata and the use of ontologies and controlled vocabularies for identifying the biosamples, targets, treatments and assays. A large curation effort was undertaken to identify the appropriate ontologies to use and the specific term identifications for biosamples for the current and past ENCODE projects (12). For more complex data searches, queries can be built using the fields from the schemas of our metadata (https://www.encodeproject.org/help/rest-api/). Although these search features can be used on any of our search pages for any of the objects (antibodies, biosamples, etc.), the primary access to the data is through the assay page. The results on this page represent an experimental collection of replicates. At any point in the searching and filtering process, the selected metadata can be downloaded in table format using the ‘Download’ button.

### Experiment pages

**Figure 3.**
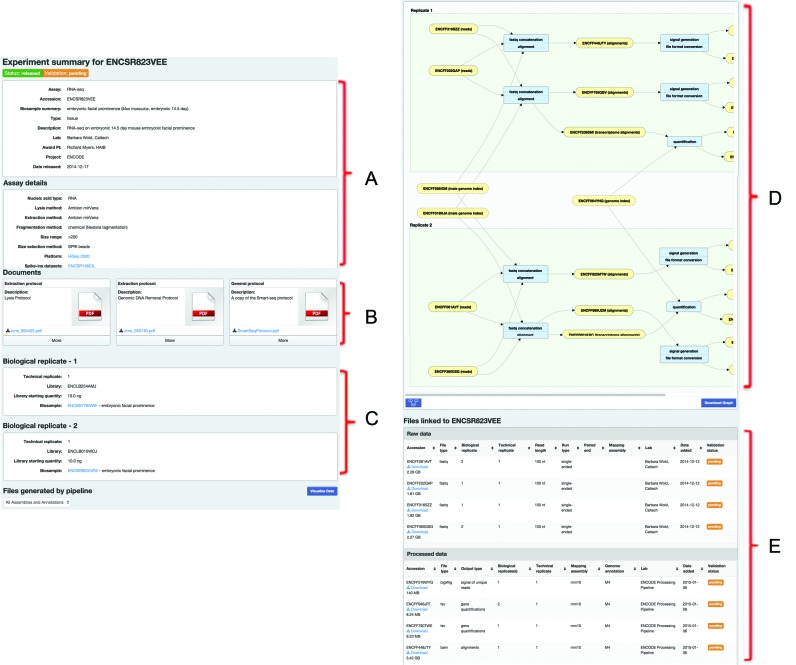
The ENCODE Portal Experiment Summary page. (**A**) Overview and details of the experiment; what assay was performed in what species using which biosamples. (**B**) Array of protocol documents (usually PDF files) describing the intricate details of experimental techniques performed. (**C**) Replicate structure indicating the number of biological and technical replicates with links to specifics about the growth or procurement of the sample. (**D**) The interactive graph of the file relationships with the software and pipeline provenance. Clicking on nodes of the graph will change the details displayed in the details section. (**E**) File listing of all files associated with the experiment with details and download links.

For any one of these replicated experimental sets, there is a dynamically generated page that collects all of the experiment's details and data. This page has five sections: the assay details, the protocol documents, replicate details, the files list and (if available) an interactive graph displaying the provenance of the data files. The assay details and the protocol documents provide a mixture of structured and unstructured metadata to fully contextualize the experiment and provide any important details required for interpretation and reproduction (Figure 3A and B). The replicate section is intended to give insight into the replicate structure of the experiment (Figure 3C). The standard replicate structure for most ENCODE experiments is two biological replicates per experiment (15). However, the portal is flexible enough to handle variation in the replicate structure. In addition, the biosample links in the replicate section, provide further details about donors, strains, growth or tissue excision details and treatments. If a cell line includes some type of transgenic construct or other genetic alterations, those details and protocols can also be found through the biosample page. The files list section has the raw data separated from any processed data associated with the experiment (Figure 3E). Details like the sequencing read length and run-type are associated with the raw data. Details such as which assembly and genome annotations were used during the generation of those processed files are found with those files in the processed data subsection. Any individual file can be downloaded from this page. When associated pipeline metadata are present for the data files, the pages additionally have a dynamically-generated graph that maps out the file relationships along with links to software and pipeline details that lead from one file to the next (Figure 3D). This graph includes all of the related files in the current experiment set and and any contributing files such as those from controls or reference sequence files. Each node in the graph is interactive and provides further details about the file or processing step and the graph structure allows a user to track the software provenance of each file. In addition to these five sections which allow context for the metadata and structure of the experiment, there is a ‘Visualize Data’ button that will create UCSC Genome browser tracks via ‘track hubs’ (16) for appropriate files.

### Dynamic track hubs

**Figure 4.**
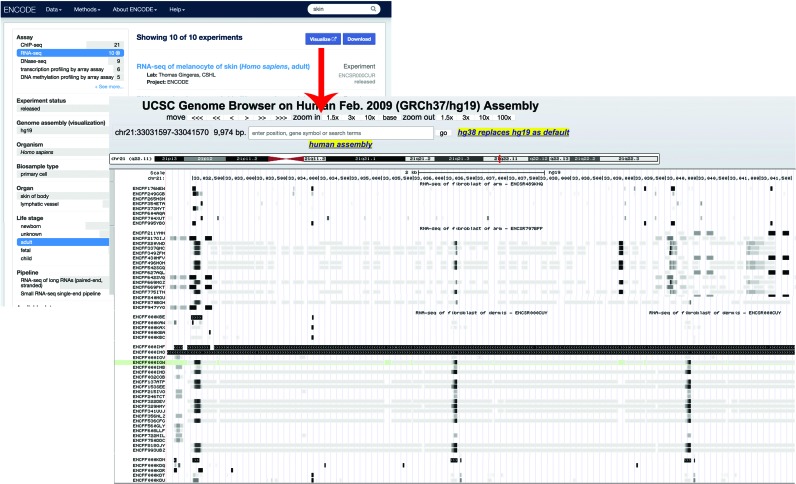
The Visualize button on the Assay search page and the individual experiment page generates a UCSC Genome Browser track hub of all appropriate files with labels generated from the metadata.

The UCSC Genome Browser provides a mechanism called a track hub to remotely host genomic data for integration into the Genome Browser's visualization tools. The majority of the released experiments at the ENCODE portal have visualizable signal or annotation files aligned to genomic coordinates in either bigWig or bigBed format (the primary file formats supported in the UCSC Genome Browser track hub feature) (17). When the user selects the Visualize Data button, all files of these formats are included in an automatically generated track hub that is immediately launched in the Genome Browser (Figure 4). These track hubs automatically includes a summary of the metadata describing the assay based on the relationship between the file and the experimental metadata. These summaries include items such as the target of the antibody in a ChIP-seq assay, a summary of the biosample and biological replicate number. This feature allows the user to easily integrate ENCODE data with their own custom tracks or the many reference tracks available at the Genome Browser. As well as being on the individual experiment page, this feature is available on the Assay search page. Once the user has filtered the list to a reasonable subset (currently <500), the ‘Visualize’ button will appear and a track hub including all appropriate files from every experiment on the list is assembled.

## FUTURE WORK

Here, we have described the new features of the ENCODE Portal: improved access to the experiment standards and methods developed by the ENCODE Consortium, metadata-driven searches to find relevant datasets and visualization of only relevant datasets. The ENCODE Portal will be continuously updated with new experimental data, processed data and analysis metadata along with enhanced searching and visualization features as the project continues. Specifically, we are planning on introducing the concept of series organization of experiments into our Assay page. With this feature, we will be able to include a collection of assays in a series of time points or differentiation stages in the faceted search along with replicated experimental sets. And to further improve searching, we are implementing ways to search the data files themselves for genomic regions, in order to direct the scientific community to relevant assays based on whether a binding site is present near a gene of interest or a gene is expressed in a specific tissue.

In addition to providing improved access to the ENCODE data, the ENCODE Portal can serve as a gateway for other genomic projects. We are currently integrating metadata and data from other epigenetics projects into the site. Specifically we will be hosting the data of the recently funded Genomics of Gene Regulation project (http://www.genome.gov/27561317), the published datasets of the modENCODE integration papers (18–20) and Roadmap Epigenomics Mapping Consortium (http://www.roadmapepigenomics.org/) (21). This will allow searching across projects for related assays. The basic framework for the site was built to allow complex relations of experimental metadata and it is made available for other projects. It is currently being adopted by the Clinical Genome Resource (ClinGen) (22).

We have worked with GEO at NCBI to map our biosample schema to their Biosample standard (www.ncbi.nlm.nih.gov/biosample) (11,23). Currently, we are working with a method to not simply import these data into their database, but also to periodically synchronize the two databases to provide access to the most up-to-date data produced by the ENCODE consortium to the larger scientific community.

## CONTACT INFORMATION

General questions about the ENCODE data portal should be directed to the encode-help@lists.stanford.edu mailing list. For any specific questions about laboratory details or methods, the producing laboratory information is available on each experimental page and in the metadata for each file. All of the source code used for the portal website is open source and available through our repository hosted at GitHub (www.github.com/ENCODE-DCC/). You can sign up for an ENCODE announcement mailing list at https://mailman.stanford.edu/mailman/listinfo/encode-announce. Data and portal updates are available on our news page at www.encodeproject.org/news/. You can also follow us on twitter at @encodedcc.

## DISCLAIMER

*Conflict of interest statement*. Some authors receive royalties from the sale of UCSC Genome Browser source code licenses to commercial entities.
